# EHMTI-0089. Relationship of sleep bruxism and migraine

**DOI:** 10.1186/1129-2377-15-S1-D32

**Published:** 2014-09-18

**Authors:** M Kato, J Saruta, M Takeuchi, H Igarashi, T Kawata, K Tsukinoki

**Affiliations:** 1 Department of Oral Sciences Division of Orthodontics, Kanagawa Dental University, Yokosuka, Japan; 2 Department of Oral Sciences and Research Institute of Salivary Gland and Health Medicine, Kanagawa Dental University, Yokosuka, Japan; 3 Department of Internal Medicine, Fujitsu Clinic, Kawasaki, Japan

## Introduction

In recent years, patient complaining of headache is increasing in the field of dentistry. In addition, when the dentist examine patients with a diagnosis of migraine, there are many cases with malocclusion. Therefore, we focused on the relationship of malocclusion and migraine.

## Aims

The current situation is treatment with drug primarily. The purpose of this study was malocclusion and migraine to look into the relationship of bruxism in particular.

## Methods

We studied 40 female patients with migraine (mean age 42 years) attending a headache clinic. Headache diagnosis was based on the International Classification of Headache Disorders;2nd Edition(IHCD-2).We applied a questionnaire about dental history, bruxism, TMD symptoms, and general condition, took face bow, and impressions of their occlusion. In addition, we made BruxChecker for each person and measured the classification of occlusal contact pattern. We compared these data with 35 healthy controls (mean age 39 years).This study was approved by the Ethics in Research of the Kanagawa Dental University.

## Results

We found significant differences in relationship between incisors and molars with migraine group (P value 0.05). The questionnaire also showed statistically significant differences about clenching and bruxism (P value 0.05).

## Conclusions

In this study, as one of the causes of migraine, I considered bruxism and clenching are involved. In particular, we observed that the molar is related to bruxism of migraine group . By analyzing detail on these results, we will consider the relevance of malocclusion and migraine.

No conflict of interest.

